# Supplementary material for: Conditional chemoconnectomics (cCCTomics) as a strategy for efficient and conditional targeting of chemical transmission
Source: eLife. 2024 Apr 30;12:RP91927. doi: 10.7554/eLife.91927 (PMC11060718; doi:10.7554/eLife.91927)
Supplement: Figure 3—source data 3. [file elife-91927-fig3-data3.zip › Figure 3 source data3 part 1.zip!/Figure 3-Source data 3-part1/1081-1-CRISPResso_Report_8zosk6yj.zip!/CRISPResso_on_1081_4/1a.Read_barplot.pdf]

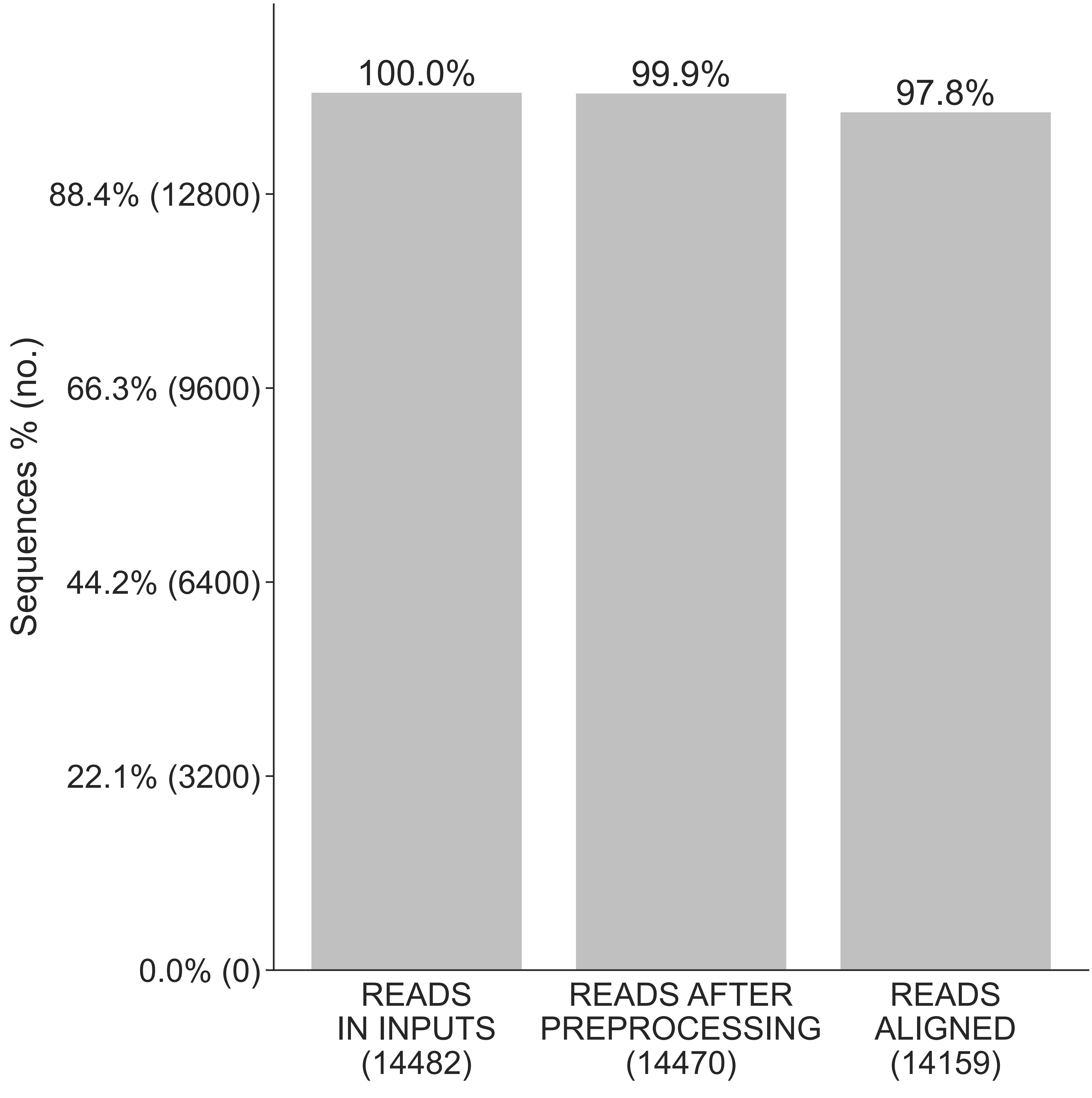
Sequences % (no.)
88.4% (12800)
66.3% (9600)
44.2% (6400)
22.1% (3200)
0.0% (0)
100.0%
99.9%
97.8%
READS IN INPUTS (14482)
READS AFTER PREPROCESSING (14470)
READS ALIGNED (14159)
